# Snapshots of the Fragmentation for C_70_@Single-Walled Carbon Nanotube: Tight-Binding Molecular Dynamics Simulations

**DOI:** 10.3390/ijms22083929

**Published:** 2021-04-10

**Authors:** Ji Young Lee, Changhoon Lee, Eiji Osawa, Jong Woan Choi, Jung Chul Sur, Kee Hag Lee

**Affiliations:** 1 Department of Chemistry, Nanoscale Sciences and Technology Institute, Wonkwang University, Iksan, Jeonbuk 54538, Korea; chemljy@hanmail.net; 2 Max Planck POSTECH Center for Complex Phase of Materials, Pohang University of Science and Technology, Pohang 37673, Korea; 3 Nanocarbon Research Institute, Shinshu University, Ueda, Nagano 386-8567, Japan; osawa@nano-carbon.jp; 4 Department of Semiconductor and Display, Wonkwang University, Iksan 54538, Korea

**Keywords:** tight-binding molecular dynamics simulation, snapshots of fragmentation, C_70_@SWCNT nanopeapods, DWCNT

## Abstract

In previously reported experimental studies, a yield of double-walled carbon nanotubes (DWCNTs) at C_70_@Single-walled carbon nanotubes (SWCNTs) is higher than C_60_@SWCNTs due to the higher sensitivity to photolysis of the former. From the perspective of pyrolysis dynamics, we would like to understand whether C_70_@SWCNT is more sensitive to thermal decomposition than C_60_@SWCNT, and the starting point of DWCNT formation, which can be obtained through the decomposition fragmentation of the nanopeapods, which appears in the early stages. We have studied the fragmentation of C_70_@SWCNT nanopeapods, using molecular dynamics simulations together with the empirical tight-binding total energy calculation method. We got the snapshots of the fragmentation structure of carbon nano-peapods (CNPs) composed of SWCNT and C_70_ fullerene molecules and the geometric spatial positioning structure of C_70_ within the SWCNT as a function of dynamics time (for 2 picoseconds) at the temperatures of 4000 K, 5000 K, and 6000 K. In conclusion, the scenario in which C_70_@SWCNT transforms to a DWCNT would be followed by the fragmentation of C_70_, after C_70_, and the SWCNT have been chemically bonding in the early stages. The relative stability of fullerenes in CNPs could be reversed, compared to the ranking of the relative stability of the encapsulated molecules themselves.

## 1. Introduction

The formation of a closed C fullerene by creating curvature through the fusion of 12 pentagon rings of carbon has been the special topic of several studies [1]. A single nanotube can be formed by wrapping and connecting the graphene sheet [2]. Carbon nanotubes are valuable materials that have excellent physical and chemical properties such as high electrical conductivity, thermal stability, mechanical strength, aspect ratio, and surface area. These unique features of carbon nanotubes (CNTs) enable the development of innovative technologies and materials that can be used in various applications such as food, energy, and the water industry. The complex relationship between these resources (water, energy, and food) requires a sustainable, integrated approach for ensuring sustainable energy production as well as the security of water and food [3]. 

One of the most prominent features of nanocarbons (e.g., fullerenes, carbon nanotubes, and graphene) is their ability to encapsulate atoms, ions, molecules, molecular ions, nanowires, or nanoribbons in empty spaces within their structures [4].

The well-known C_60_ molecule is highly (almost spherically) symmetric. Therefore, a C_60_ molecule encapsulated in a CNT remains particularly mobile, both orientationally and translationally, until low temperatures, as predicted from theory [5] and confirmed by experiments [6]. In addition, when using tight-binding molecular dynamics, the decomposition process for C_60_@SWCNT [7] and (C_60_)_2_@SWCNT [8] was studied in which the decomposition process was at 1000 K intervals in the temperature range of 4000 to 6000 K. Using the internal space of the SWCNT as a nanometer-scale reaction chamber, double-walled carbon nanotubes (DWCNTs) forming secondary tubes inside the SWCNT were observed, which confirmed that the temperature excitation can be used to overcome the activation barrier for the inner tube formation [9]. The C_60_ molecules inside the SWCNT can remain unchanged at up to 800 °C without de-doping. Further heating to about 1200 °C induces cohesion between the C_60_ molecules, resulting in the molecules forming tubular structures. The annealing binding can potentially produce double-wall carbon nanotubes in a macroscopic amount. The interest in studying this material with macroscopic spectroscopy has been gathered by showing that Raman spectroscopy can be performed on DWCNTs [10]. Using equilibrium molecular dynamics simulations, the thermal conductivity of CNPs was described as the interaction between the nanotubes and the encapsulated fullerene particles, and the possible margin of movement between the fullerene particles. It was shown to decrease with the coalescence of encapsulated fullerene molecules [11]. 

It was claimed that the conversion of C_70_@SWCNT into double-walled carbon nanotubes was more efficient than the corresponding conversion of C_60_@SWCNT due to the higher sensitivity to photolysis of the former [12]. Thus, it is interesting to see the snapshots of fragmentation of C_70_@SWCNT to understand the formation of double-walled carbon nanotubes grown by thermal treatment of peapods (the thermal annealing). We have a lot of interest in cases where molecules are inserted inside of carbon nanotubes, and it would be very interesting and important to know how C_70_ molecules behave in these nanotube structures. 

The decomposition or fragmentation of C_70_ fullerenes contained within a nanotube can be studied by tight-binding molecular dynamics (TBMD) analysis. Moreover, simulation studies of the interaction between carbon nanotubes and C_70_ molecules at different temperatures could be helpful for understanding the thermal conductivity indirectly. The exact mechanism behind the formation of carbon materials is difficult to elucidate because the control of many of the experimental conditions is still challenging. Therefore, it would be very interesting to study the fragmentation behavior of CNPs in an indirect way to gain an understanding of the mechanism underlying DWCNT formation. In this study, we applied TBMD calculations to study the dynamics fragmentation behavior of CNPs. The accuracy of our calculations depended on our selection of the correct empirical tight-binding parameters for both the molecule dynamics simulations and the empirical TBMD method for electronic band structure calculations. Here, the carbon system parameters have been used to reproduce the universal binding energy curves of various stages obtained based on the computation of the first principles [13]. This potential has been successfully applied to various carbon systems [14,15,16,17]. In addition, the fragmentation of C_60_ [18] and C_70_ [19] clusters, C_20_ isomer clusters [20], and C_60_@SWCNT [7], and (C_60_)_2_@SWCNT [8] has already been studied using these TBMD parameters. 

## 2. Results and Discussion

### 2.1. Geometry of C_70_, SWCNT, And CNP 

**Figure 1:**
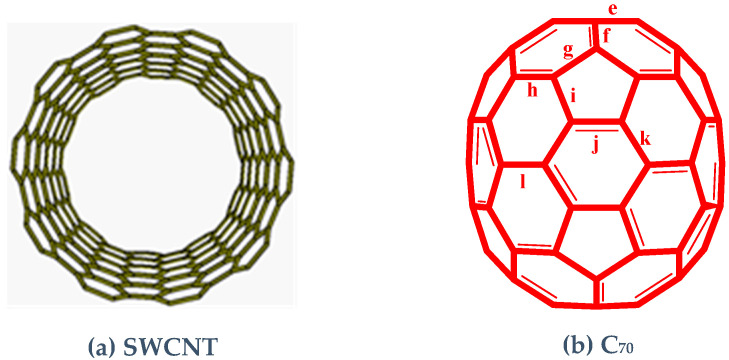
Structures of a single-walled carbon nanotube (SWCNT) and C_70_ fullerene molecule (length unit: Å). (**a**) Single-walled carbon nanotube (SWCNT) is composed of a chirality of 10 × 10, diameter of 13.551, unit cell length of 14.989, and 240 atoms in a unit cell. The bond lengths of SWCNT were 1.431 Å, respectively. (**b**) In C_70_, the bond length of **e, f, g, h, i, j, k**, and **l** is 1.452, 1.397, 1.448, 1.389, 1.449, 1.434, 1.432, and 1.471 Å, respectively.

The bond distances of SWCNT with the TBMD simulations [13,21,22] for the ground state are shown in Figure 1a. The atomic structure of the optimized C_70_ molecule shown in Figure 1b was obtained using Becke’s three-parameter hybrid method and Lee-Yang-Parr exchange correlation function theory (B3LYP) [23,24,25,26,27,28] with the electron based set 6–31G (d, p).

**Figure 2:**
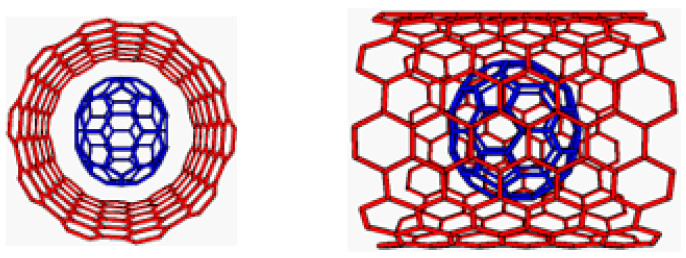
Model of a carbon nano-peapod with C_70_ fullerene encapsulated within a single-walled (10, 10) carbon nanotube.

The SWCNT in Figure 1a is composed of a chirality of 10 × 10, diameter of 13.551 Å, unit cell length of 14.989 Å, and 240 atoms in a unit cell. The bond lengths were 1.431 Å. In Figure 1b, C_70_ has eight kind of the bond lengths of **e**, **f**, **g**, **h**, **i**, **j**, **k**, and **l**, which is 1.452, 1.397, 1.448, 1.389, 1.449, 1.434, 1.432, and 1.471 Å, respectively. In the TBMD simulations, the initial CNP structure was obtained by matching the center of gravity of C_70_ with the center of gravity of the nanotube (as shown in Figure 2). Moreover, the C_70_ and SWCNT structures used were obtained using the Becke’s Three Parameter Hybrid Functional Using the LYP Correlation Functional (B3LYP) and TBMD methods, respectively (as shown in Figure 1).

### 2.2. Distance Distribution Function of Adjacent Carbon Atoms in a CNP

**Figure 3:**
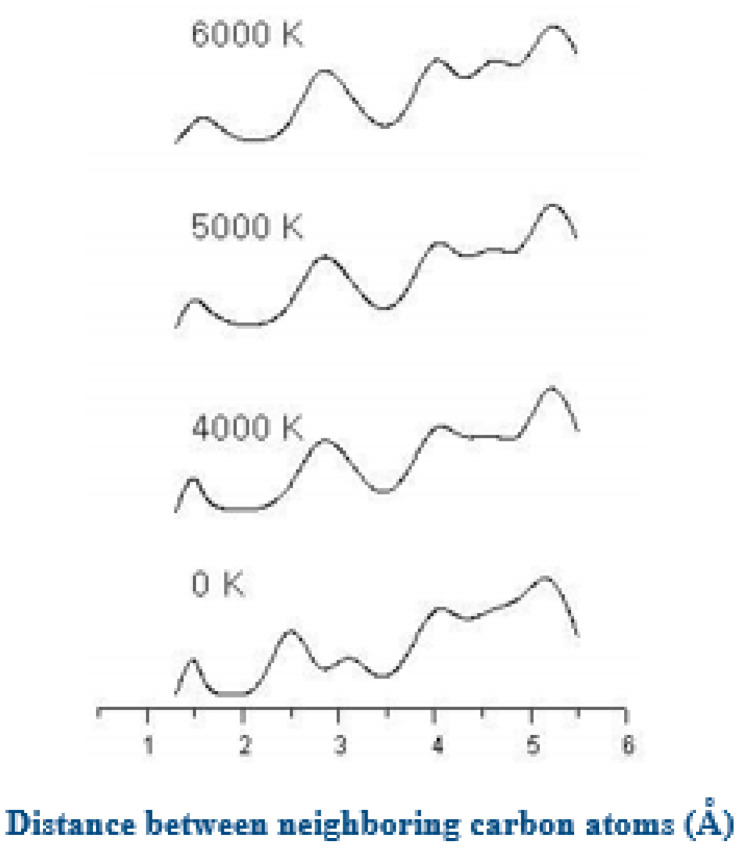
Radial distribution function of the distance (Å) between neighboring carbon atoms in the carbon nano-peapod structure at various temperatures.

Figure 3 shows the distance distribution function of adjacent carbon atoms in a CNP structure consisting of carbon nanotubes with C_70_ fullerenes inserted, at temperatures of 0, 4000, 5000, and 6000 K, respectively. 

**Figure 4:**
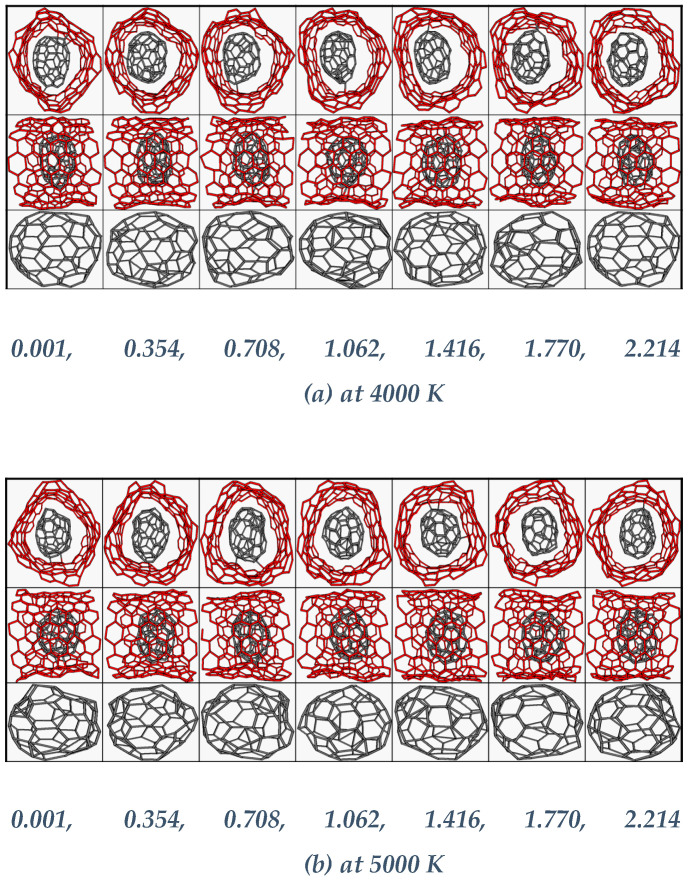

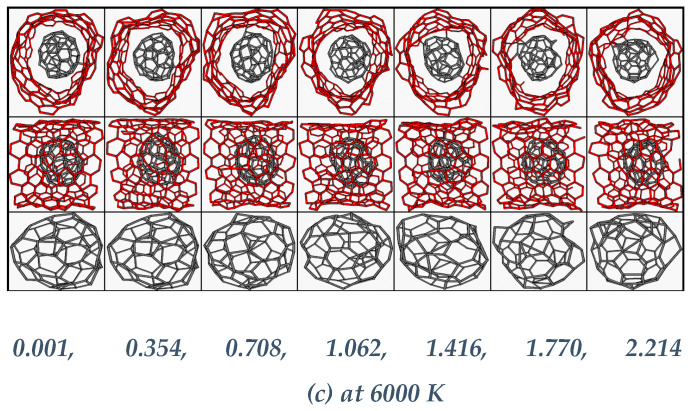
Snapshots of the fragmentation process for the carbon nano-peapod (CNP) at 4000 K in (**a**), 5000 K in (**b**), and 6000 K in (**c**), respectively. Here, the simulation time (unit: picosecond) is of the last row of snapshots in a regular sequence.

In addition, by using the snapshots in Figure 4a–c, it could be shown that we could obtain a successful formation yield of higher DWCNTs at C_70_@SWCNTs than C_60_@SWCNTs in Reference [7]. In previously reported experimental studies, a yield of DWCNT at C_70_@SWCNT is higher than C_60_@SWCNT due to the higher sensitivity to photolysis of the former. Thus, C_70_@SWCNT is more sensitive to both thermal and photolysis reactions than C_60_@SWCNT. The passage of thermal conductivity in the CNP was mostly transmitted through C_70_ rather than CNT, which was consistently the finding of a previous study [11].

We should be keeping in the mind the point of view that the stability of the nano-peapod is different from the stability of an encapsulated molecule itself such as when observing which C_70_ is more stable than C_60_ in the point of energetics. It is in line with the reports that a combination of electronic and steric requirements inside fullerenes and nanotubes significantly changes the stereochemical properties of even relatively simple molecules in comparison with their free state [23], in which, by using neutron spectroscopy, there are some couplings between vibrational modes and whole molecule mobility of the confined fullerene in C_60_@SWCNT [24], and that the polymerization of fullerenes and the chemical interaction between the fullerenes and the tube wall is observed in the tube filled with fullerenes in the bending of 270 degrees by using the empirical and the quantum mechanical methods [25]. 

In the future, we hope to apply our approach for real-world applications, such as those involving fullerenes and multi-wall carbon nanotubes. We would like to extend our approach to simulations, first for understanding fragmentation and formation of complexes comprised of nano-diamonds and graphene, and for the decomposition of fullerenes and nanotubes including the effect of the honeycomb flat size around the defect, as reported in skeletal rearrangements of fullerene [26].

## 3. Materials and Methods

### 3.1. Tight-Binding Molecular Dynamics Simulations

In the TBMD scheme, the total Hamiltonian is written as follows:

(1) $$E=\sum^{}\frac{P_{I}^{2}}{2m}+\overset{occupied}{\underset{n}{\sum }}\langle \psi_{n}\left|H_{TB}\right|\psi_{n}\rangle +U_{rep.}$$

(2) $$U_{rep}=\underset{I}{\sum }f\left[\underset{J}{\sum }\varphi \left(r_{I,J}\right)\right]$$

The first, second, and third terms represent the kinetic energy of the carbon atoms, the electronic energy calculated from the Hamilton *H_TB_* bound by parameters, and the short-range repulsive energy *U_rep_*, respectively, where ϕ(*r_I,J_*) is the pairwise potential function. The above parameters and functions were fitted to the electronic band structure and binding energy results obtained by first-principle calculations for various crystalline carbon phases. 

If the total energy calculation method is constructed in Tight-Binding (TB), the next step in the TBMD simulation is to calculate the force per atom. This can be achieved by taking a derivative of the total energy for each atom, as follows:

(3) $$F_{i}=ma_{i}=-\frac{\partial E_{tot}}{\partial r_{i}}=-\overset{occupied}{\underset{n}{\sum }}\langle \psi_{n}\left|\frac{\partial H_{TB}}{\partial r_{i}}\right|\psi_{n}\rangle -\underset{I<J}{\sum }\frac{\partial U_{r_{IJ}}}{\partial r_{i}}$$

The first term here is called the Hellman-Feynman force and the second term comes from the repulsive pairwise potential [21]. These forces are supplied to the molecular dynamics simulations, and the 3N coupled secondary differential equations are numerically solved through the Gear algorithm [22]. Since this method uses the valence electrons of atoms, its computational efficiency is improved compared with that of ab initio calculations, which is a very important factor in practical calculations [13].

**Scheme 1:**
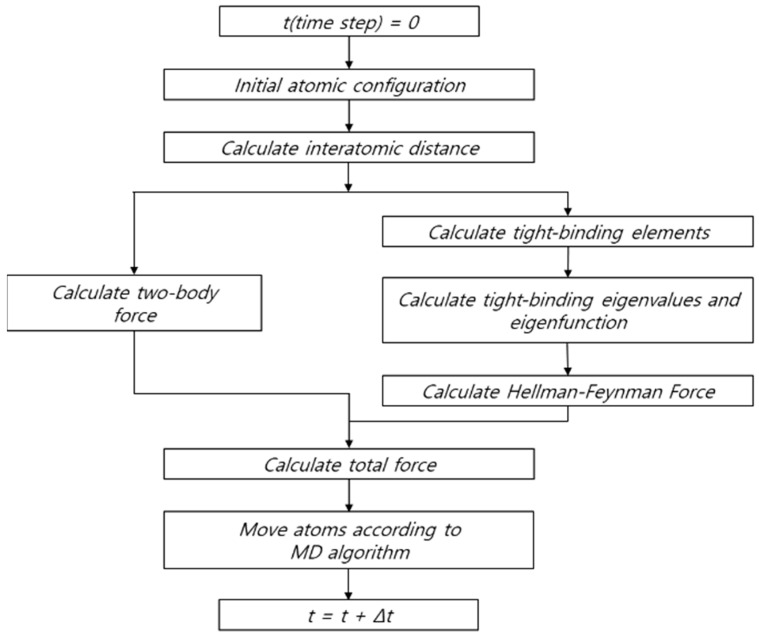
Flow chart of the tight-binding molecular dynamics calculations.

Scheme 1 shows the TBMD flow chart for the calculations. We use 7.08 × 10^−16^ s as a one-time step. In the total energy calculations, we used 10^−3^ eV as a convergence criterion for the energy surface of the microcanonical ensemble at room temperature.

The TBMD simulations were performed in thousands of time steps to ensure equilibrium. The system was set to rerun 1500-time steps at a given temperature with a typical temperature control method (canonical ensemble), and then 1000-time steps at the microcanonical ensemble. The equilibrium structures, heat capacities, bond distributions, assembly energies, total energies, and charge distributions were then calculated in an average of more than 3000-time steps at a given temperature.

### 3.2. Hybrid Density Functional Calculations

In this study, hybrid density functional theory, using Becke’s three-parameter hybrid method combined with Lee-Yang-Parr exchange-correlation functional theory (B3LYP) [27,28,29,30,31,32], was applied to optimize the geometries of the C_60_ molecule, as shown in Figure 1b. It also used the electron basis set 6–31G (d, p) [31]. We used the Gaussian 2003 B.04 package suite [32] to fully optimize the geometry of C_70_, by applying the convergence criterion with tight optimization and an ultrafine pruned (99,590) grid.

## Data Availability

Data available upon request.
